# Supplementary material for: Basic Guide to Multilayer Microfluidic Fabrication with Polyimide Tape and Diode Laser
Source: Micromachines (Basel). 2023 Jan 27;14(2):324. doi: 10.3390/mi14020324 (PMC9959566; doi:10.3390/mi14020324)
Supplement: Supplementary file 1 [file micromachines-14-00324-s001.zip › micromachines-2176866-supplementary.pdf]

Basic guide to multilayer microfluidic fabrication with polyimide tape and diode laser

Supplementary Information

Thana Thaweeskulchai * and Albert Schulte

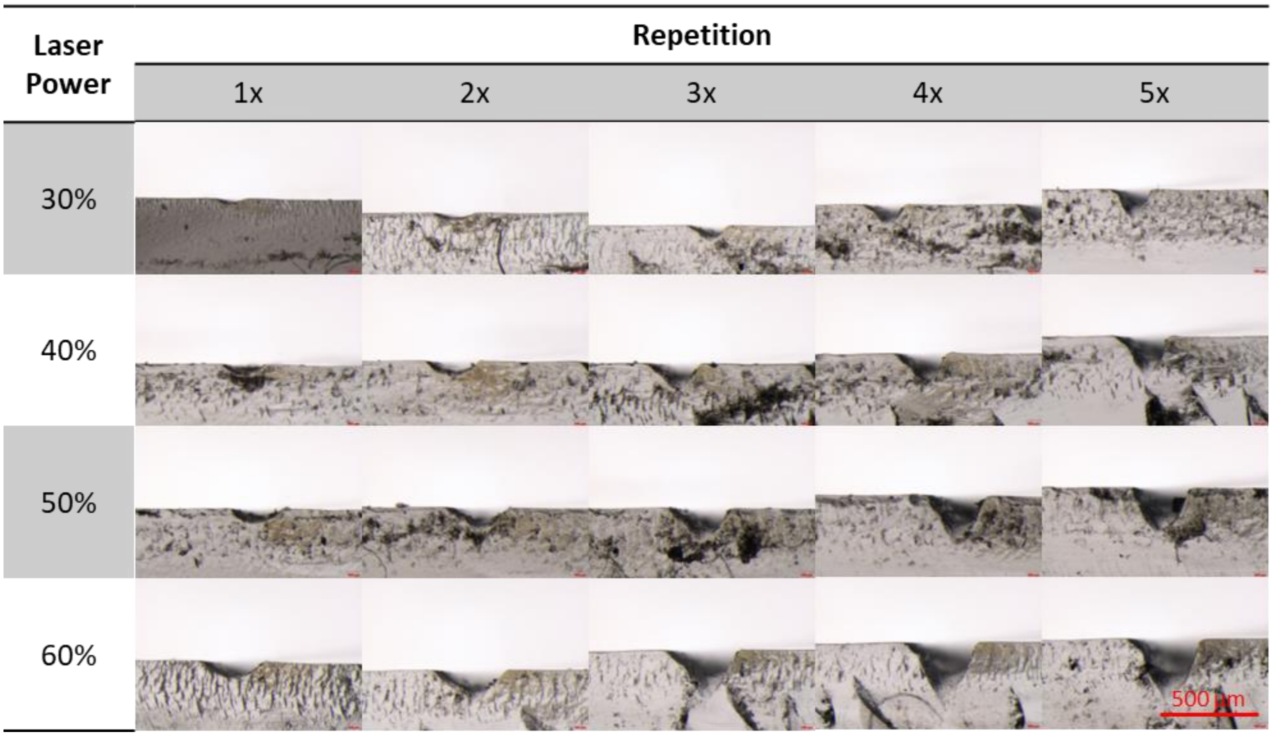

SI Figure S1. Photographs of cross-sectional profiles of microchannels fabricated with Raster single-line mode.

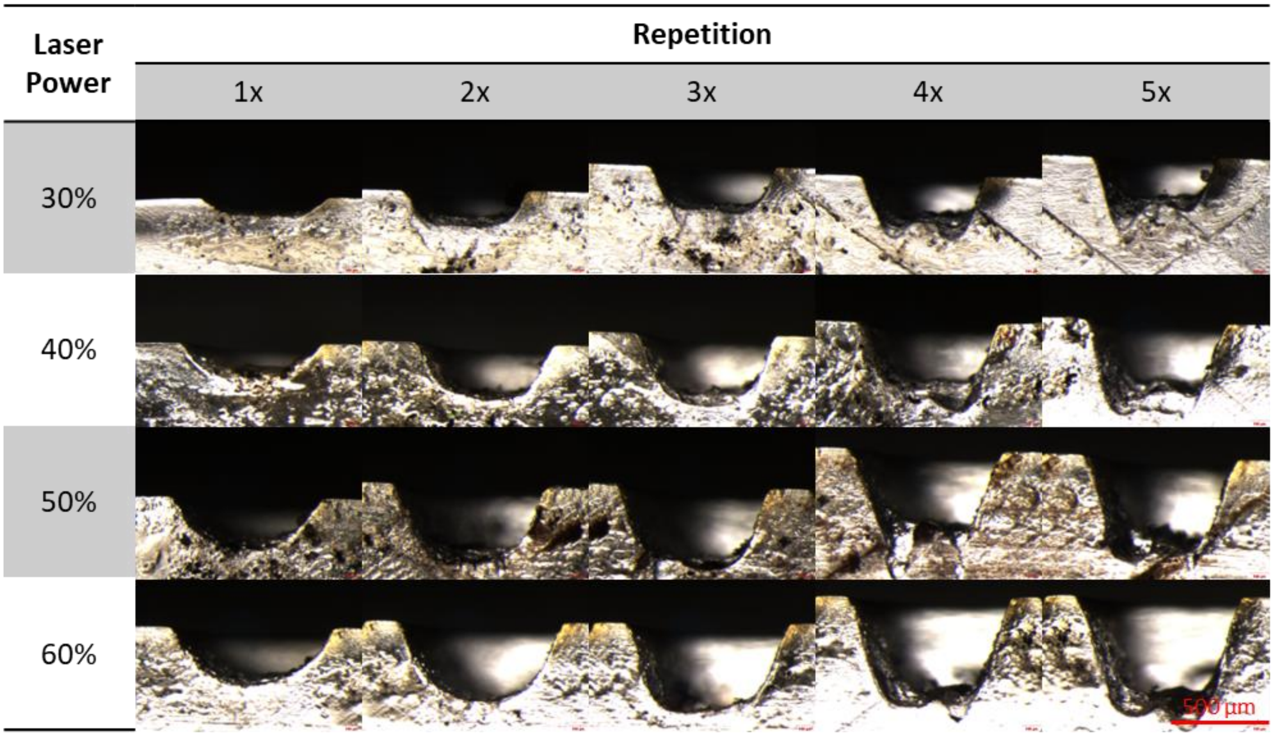

SI Figure S2. Photographs of cross-sectional profiles of microchannels fabricated with Raster 0.5mm mode.

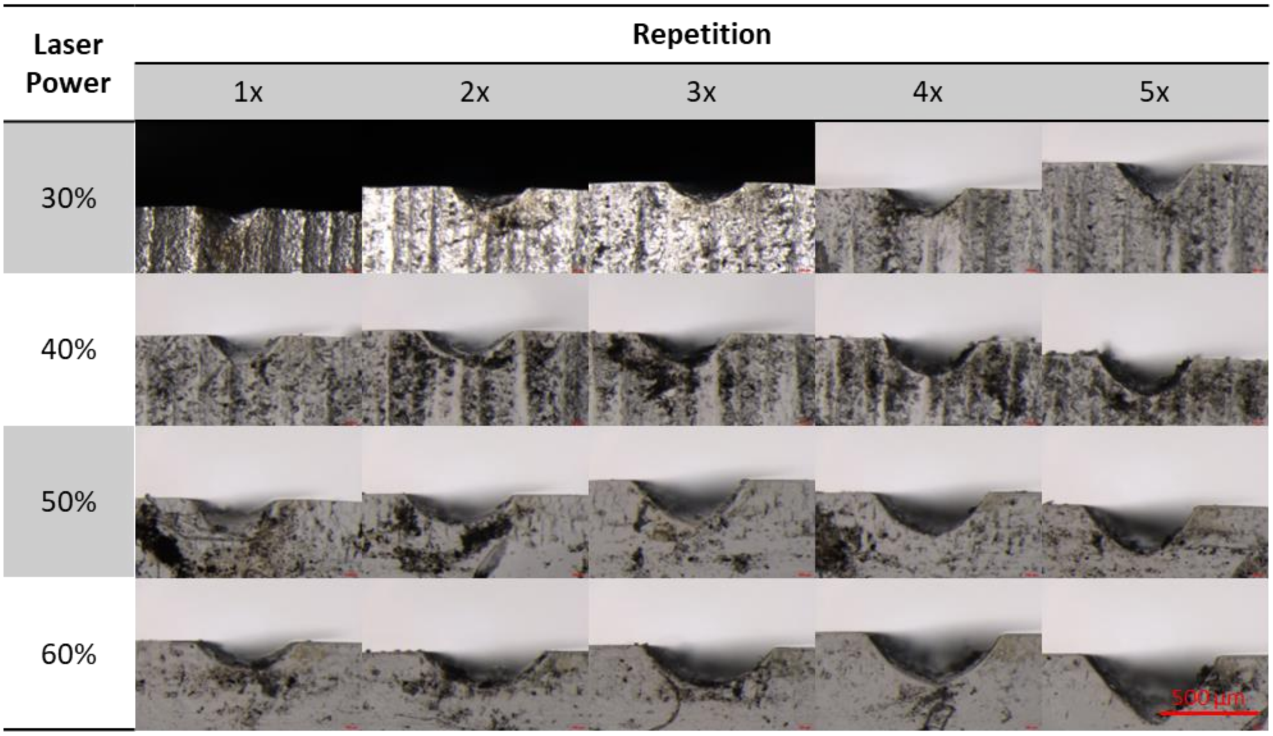

SI Figure S3. Photographs of cross-sectional profiles of microchannels fabricated with Vector single-line mode.

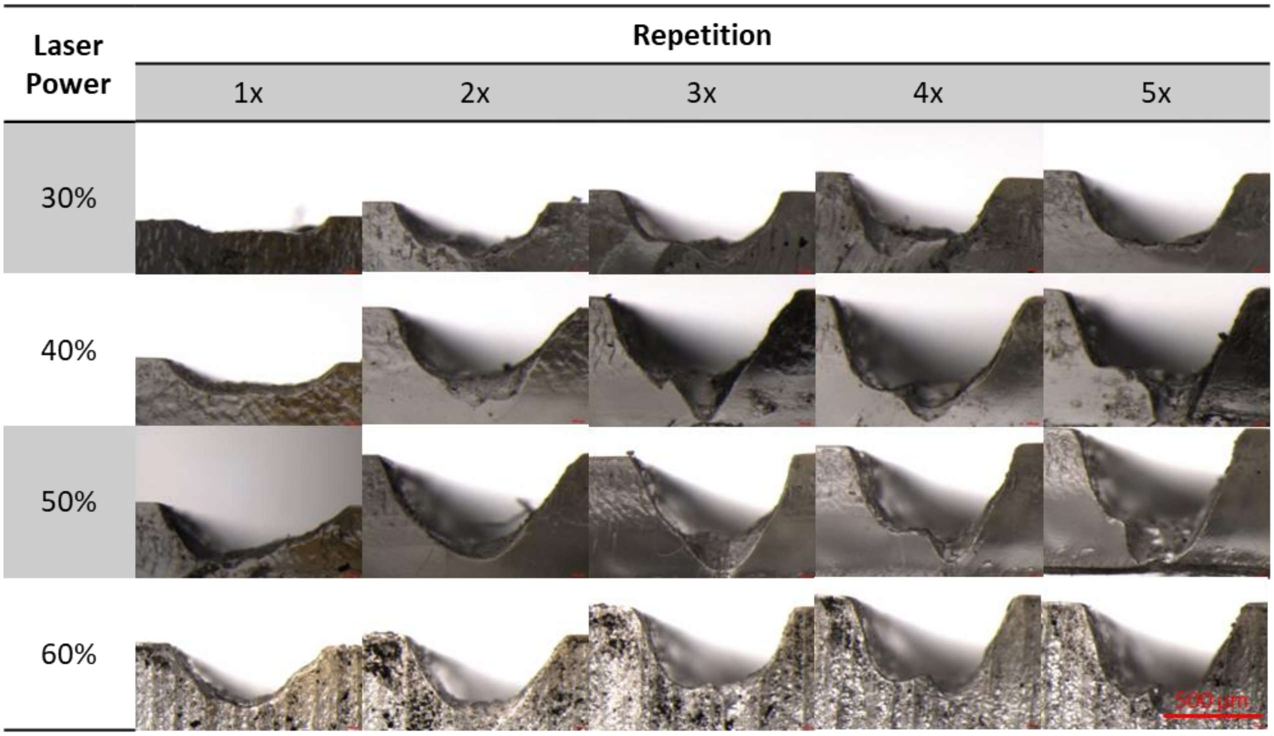

SI Figure S4. Photographs of cross-sectional profiles of microchannels fabricated with Vector 0.5mm mode.

SI Table S1. Abbreviations used.

POC	Point-of-care
SCF	Spontaneous capillary flow
SARS-CoV-2	Severe acute respiratory syndrome-coronavirus-2
CNC	Computer numerical control
PMMA	Polymethyl methacrylate
CAD	Computer-aided design
IPA	Isopropyl alcohol
